# Post-traumatic stress disorder (PTSD) in parents of children with type 1 diabetes during Covid-19 pandemic

**DOI:** 10.1186/s13052-021-01126-0

**Published:** 2021-08-26

**Authors:** C. Carducci, N. Rapini, A. Deodati, V. Pampanini, S. Cianfarani, R. Schiaffini

**Affiliations:** 1 Clinical Psychology Unit – Bambino Gesù Children’s Hospital, IRCCS, Rome, Italy; 2 Diabetes Unit - Bambino Gesù Children’s Hospital, IRCCS, Piazza S Onofrio, 4, 00165 Rome, Italy; 3 Tor Vergata University, Rome, Italy; 4 Department of Women’s and Children’s Health, Karolinska Institutet and University Hospital, Stockholm, Sweden

**Keywords:** Pediatric diabetes, Post-traumatic stress disorder, SARS-CoV-2, Covid-19

## Abstract

**Introduction:**

The Post-traumatic Stress Disorder (PTSD) is a group of persistent psychological and physiological symptoms due to a traumatic, severe, event. Only few studies focused on the effects of Covid-19 on psychosocial outcomes in children with Type 1 Diabetes (T1D) and their parents.

**Aim of the study:**

The aim of this study was to evaluate the presence PTSD in parents of children with T1D during COVID-19 pandemic lockdown.

**Patients and methods:**

In the period between March and May 2020 we submitted the “Impact of Event Scale – Revised” (IES-R) questionnaire to the parents of 34 children with Type 1 Diabetes, asking them to express their emotions about the ongoing Covid-19 pandemic.

**Results:**

A total of thirty mothers (mean age 43.0 ± 4.2 years) and 25 fathers (mean age 45.6 ± 5.9 years) participated in the survey and completed the questionnaires. 29.1% of parents had a score that allows to define a clinically relevant level of PTSD; ten mothers and 6 fathers had a PTSD clinically relevant score, corresponding, respectively, to 28.4 and 24% of total mothers and fathers. Finally, mothers and fathers, both express PTSD symptoms mainly in the form of intrusion and hyperarousal.

**Conclusions:**

The present study confirms a high prevalence symptoms related to PTSD in mothers and fathers of children with Type 1 Diabetes. We believe that psychosocial outcomes of the COVID-19 pandemic should be taken into account in the planning of the next future assistance for children with T1D.

## Introduction

Type 1 diabetes (T1D) is one of the most common chronic diseases in infancy [1] and the most frequent endocrinopathy in childhood. Pediatric diabetes management requires complete parental dedication and involvement and exposes the whole family to daily challenges, related to the need for frequent blood glucose monitoring, intensive insulin therapy and specific dietary indications [2, 3].

The Post-traumatic Stress Disorder (PTSD) is a group of persistent psychological and physiological symptoms due to a traumatic, severe, event. As reported in the Diagnostic Statistical Manual (DSM) of Mental Disorders (4th and 5th editions) a post-traumatic event is classified as a life-threatening event and it is perceived as a risk for physical and mental integrity. PTSD belongs to a category, called “Trauma- and Stressor-Related Disorders” [4, 5].

The acute onset and the chronic diabetes management are, both, related to PTSD. Studies have found that a diabetes diagnosis in a child is associated with psychological distress among parents. In particular, a study of newly diagnosed children with diabetes showed that nearly one-fourth of mothers and fathers met criteria for PTSD 6 weeks postdiagnosis [6].

Usually, parents of children with diabetes report initially an acute stress, strictly related to diabetes diagnosis. This psychological status can be followed by a chronic, persistent stress, essentially related to traumatic aspects of medical treatment.

Sars-Cov-2 (Covid-19) infection has first been reported in Wuhan, China in December 2019 and so far, it affected about one hundred and ten millions patients in more than 220 countries and has caused more than 2,400,000 deaths [7, 8]. In Italy lockdown of the entire country has been claimed in March 9th, 2020 in order to prevent/reduce the spread of pandemic. It lasted until mid-May and has forced the entire Italian population to a considerable psychological distress [9].

Indeed, the lockdown and the fear of Covid-19 pandemic greatly influenced our society. The majority of scientific studies have focused on biochemical aspects or on the interaction between the virus and existing diseases. Medical effects have received the most attention, whereas only few studies focused on the Covid-19 pandemic direct effect on mental health and psychosocial attitude.

Pediatric T1D is a very fragile context, in which a destructing event, as was the lockdown in the acute phase of the pandemic, can induce emotional adaptation disorders such as PTSD. It, therefore, seems important to underline the psychological relatedness of the SARS-COV2 infection with its possible consequences. In particular, the COVID-19 outbreak would result in higher levels of psychological distress among people and families managing with T1D.

### Aim of the study

The aim of this study was to evaluate the presence PTSD in parents of children with T1D during COVID-19 pandemic lockdown.

## Patients and method

In the period between March and May 2020, we consecutively interviewed the parents of 34 children with T1D followed at the Diabetes Unit of the Bambino Gesù Children’s Hospital, Rome – Italy. Patients involved in the study were all affected by Type 1 Diabetes, with a diabetes duration of at least 1 year and a reference age range between 1 and 18 years; in order to avoid the psychological distress related to the onset of the disease, we excluded families of patients with recent diagnosis. The interview was done in a tele-assistance context, which was the prevailing assistance mode during the lockdown period. During the lockdown period the center performed “in presence visits” only for emergency issues and those families were excluded from the interview. The study was conducted according to the Declaration of Helsinki. Participants provided informed consent. Potential conflict of interest do not exist. Patients’ data are available and the paper did not request access to research funds.

We submitted the “Impact of Event Scale – Revised” (IES-R) questionnaire to the partecipants, asking them to express their emotions about the ongoing Covid-19 pandemic.

The IES-R is a self-report 24-item questionnaire, used in order to assess subjective distress caused by traumatic events. Items directly refers to 14 out of the 17 DSM-IV symptoms of PTSD. Respondents are asked to identify a specific stressful life event and then indicate how much they were distressed or bothered during the past 7 days by each “criticism” listed. Items are rated on a 5-point scale ranging from zero (“not at all”) to four (“extremely”). The IES-R yields a total score and according to the final score, partecipants are allocated in a specific range that defines the presence and the severity (mild to very severe) of traumatism.

## Results

A total of thirty mothers (mean age 43.0 ± 4.2 years) and 25 fathers (mean age 45.6 ± 5.9 years) participated in the survey and completed the questionnaires, without missing items.

The mean total scores and those relating to the “Avoidance, Intrusion and Hyperarousal” sub-scales are reported in Table 1. Mothers have a higher average score than fathers.

**Table 1:** Mean scores of the IES-R questionnaire

	Total mean score	Avoidance scale mean score	Intrusion scale mean score	Hyperarousal scale mean score
Total n.55	2.96	0.93	1.13	0.92
Mother n.30	3.15	0.96	1.16	1.32
Father n.25	2.72	0.87	1.02	1.11

**Fig 1:**
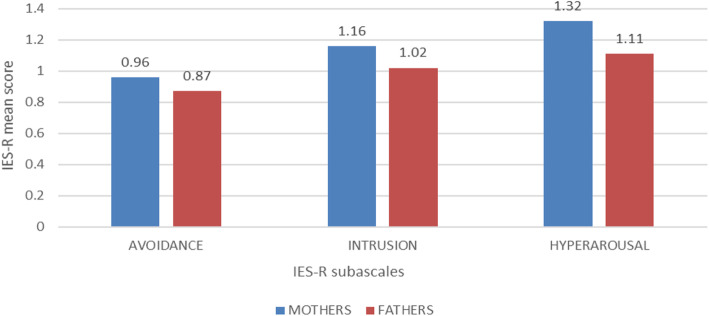
Mean PTSD scores related to Avoidance, Intrusion and Hyperarousal sub-scales

Analyzing the individual answers, the following data were highlighted:

1. Sixteen out of 55 parents (29.1%) had a score that allows to define a clinically relevant level of PTSD (raw scores above 33)
2. Ten mothers and 6 fathers had a PTSD clinically relevant score, corresponding, respectively, to 28.4 and 24% of total mothers and fathers
3. Overall, the percentage of parents with active stress symptoms is generally high in our sample; mothers and fathers, both express PTSD symptoms mainly in the form of intrusion and hyperarousal (Fig. 1)

Finally, we made a comparison analysis between parents with symptoms related to the PTSD area and those without symptoms; this analysis demonstrates a significant age difference between parents reporting high levels of stress and those reporting symptom-free status (42 vs 45,5 years).

## Conclusion

In a previous evaluation [10], we described a clinically relevant presence of trauma in a high percentage of mothers and fathers of T1D children, affecting also the Quality of Life of entire families. Also a recent review [11] on the psychosocial outcomes of the Covid-19 pandemic has highlighted how the periods of forced quarantine performed by different states worldwide have caused or at least favored the appearance of psychological, as well as physical, consequences; these include PTSD as a critical element in the management of children and adolescents affected by diabetes during this peculiar historical moment.

The present study confirms a high prevalence of PTSD symptoms in this category of patients. About a third of families of children and adolescents with diabetes experience symptoms related to the trauma area. In particular, in our present assessment mothers and fathers of children and adolescents with T1D express PTSD symptoms mainly in the form of intrusion and hyperarousal. This could have an impact on glycometabolic control and perhaps on the path of management autonomy that these patients need to achieve.

The limitation of the present study is that there is no interview to the same families with the same questionnaire at the first post-lockdown visit in presence.

In the next future it would be indeed interesting to understand if the stress symptoms were only related to the lockdown period.

In conclusion, we believe that the presence of symptoms related to PTSD should be taken into account in the planning of the assistance to be reserved for families of children and adolescents with T1D, especially in the next phase of routine recovery of diabetes management. Future studies will help to better understand whether the psychosocial outcomes of the COVID-19 pandemic will have an impact on glycometabolic control metrics.

## Abbreviations

PTSD: Post-traumatic stress disorder
T1D: Type 1 diabetes
IES-R: Impact of event scale-revised
DSM: Diagnostic statistical manual
